# Assessment of the durability of polyurea resin coatings against selected aggressive solutions in the sewage infrastructure environment

**DOI:** 10.1038/s41598-026-37921-0

**Published:** 2026-01-31

**Authors:** Barbara Francke, Hanna Michalak, Dorota Kula, Gabriela Rutkowska, Wojciech Zięba, Bartłomiej Salata

**Affiliations:** 1 Institute of Civil Engineering, Department of Civil Engineering, Warsaw University of Life Sciences-SGGW, Nowoursynowska 159, Warsaw, 02-787 Poland; 2 Faculty of Architecture, Warsaw University of Technology, Plac Politechniki 1, Warsaw, 00-661 Poland; 3 Institute of Civil Engineering, Department of Structural Mechanics, Warsaw University of Life Sciences-SGGW, Nowoursynowska 159, Warsaw, 02-787 Poland; 4 Faculty of Civil Engineering, Warsaw University of Technology, Plac Politechniki 1, Warsaw, 00-661 Poland

**Keywords:** Polyurea resin coatings, Resistance to aggressive solutions, Mechanical performance, Concrete protection, Municipal wastewater treatment plants, Chemistry, Engineering, Environmental sciences, Materials science

## Abstract

This article presents the results of a study on how selected chemical solutions affect the durability of specific properties of polyurea resin coatings used for surface protection of concrete in wastewater treatment plants. The solutions tested included sulphuric acid at 1% and 10%, phenol at concentrations of 0.1% and 1%, and urea at 3%. Three randomly chosen coatings were treated with these solutions for 7 and 28 days. After treatment, changes in appearance, weight, hardness, and tensile strength were assessed. Tests were performed using our proprietary methods and in accordance with the standards EN 868 and EN 527-3. It was observed that phenol solutions had the most pronounced negative impact on all polyurea resin coatings, causing significant soaking (up to 30%) and reductions in hardness (up to 40 Shore units) and tensile strength (up to 80%). The 3% urea solution led to a decrease in hardness (up to 17 Shore units) and resulted in notable reductions in tensile strength (ranging from 10% to 30%). Sulphuric acid solutions within the tested concentration range (1% to 10%) caused minor changes in the coatings’ functional properties, including a 10–30% decrease in tensile strength and up to a 14% reduction in hardness.

## Introduction

Durability is a crucial aspect of constructing and using all building structures, including those made of concrete, which sometimes require additional surface protection in exceptional service load conditions. Proper protection in the construction process of surfaces designed to transfer useful loads, particularly those exposed to water, moisture, and an aggressive chemical environment, is a more economical solution than later repairs of structures damaged during operation^1^. Such safeguards are required in both residential construction and industrial buildings, including farm buildings used in agri-food processing^2^. A specific example is sewage treatment plants, where these structures are additionally exposed to chemical effects. In such facilities, polyurea resins can be utilized for surface protection^3–13^. In sewage treatment plants, these products are typically used as coatings^14^, similar to waterproofing applications in industrial construction and other economic areas^15^. An important benefit of these coatings in housing and technical infrastructure is their long-lasting durability, typically ranging from 25 to 30 years and up to 50 years^16^, compared to traditional waterproofing coatings, which last about 10–20 years^3^.

Polyurea resin is produced through a chemical reaction between isocyanate and a polymer resin blend, with added pigments and substances that enhance adhesion and resistance to external factors^4]– [5^. This process, known as polymerization, creates a polymer with unique properties such as elasticity, water resistance, and chemical resistance, which are the focus of many studies^17–23^. The final product exhibits a distinctive micro-phase separation structure comprising soft and hard segments^24–26^. Expanding the range of applications and customizing polyurea for specific uses is achieved by changing its chemical structure, combining it with various nano-additives or other polymers, and maintaining proper polymerization conditions^27,28^.

Depending on the nano-additives used, the resulting polyurea composite can exhibit, for example, improved mechanical and thermal properties compared to traditional materials, enhanced corrosion resistance in environments with different chemical aggressiveness^29^, and increased fire resistance^27^. Studies reported in^30^ show that adding silver nanoparticles improves the corrosion resistance of polyurea resin coatings, especially in sodium chloride solutions (3.5% concentration). Metal fillers also enhance other properties, such as mechanical strength, electrical conductivity, and weight. Incorporating carbon fiber reinforcements (CFRP) into the composite boosts strength, stiffness, high energy absorption, and consequently, high impact or puncture resistance^31^. Oxidized ceramic fillers, on the other hand, improve mechanical strength, thermal insulation, hardness, fatigue strength, as well as resistance to corrosion and high temperatures^32^. Key properties supporting the use of polyurea resin coatings in construction include durability^9,33–40^, weathering resistance^41^, ease of application^17,42–50^, damage mitigation^51^, chemical and biological corrosion resistance^17,42–50^, good substrate adhesion, water resistance^52^, hydrophobicity^53^, abrasion resistance, aging resistance, and mechanical properties such as high tensile strength and impact resistance^27,54^, explosion resistance^55–58^, and seismic resilience^3,59,60^. Polyurea resin coatings are well-known for their excellent mechanical behaviour, high chemical resistance, and watertightness even under harsh climatic conditions^61–63^, including seawater^17^. They also feature high elasticity (with tensile tests often showing elongation at break exceeding 500%), and their crack-bridging ability greatly reduces secondary cracking and coating failure caused by substrate scratches^64^. However, natural climatic factors can lessen their durability^65–67^, making surface protection from UV radiation essential, as extensively discussed in^68^. Due to their strong adhesion to concrete, polyurea resin coatings are considered effective for surface protection^61,65^. They tend to peel off in high-strength concrete structures (fck ≥ 50 MPa)^69^. Due to their chemical resistance to various aggressive solutions, they are also used to protect concrete structures in municipal wastewater treatment plants^70^, where microbiological corrosion can cause damage to concrete. Water and sewage infrastructure elements often face severe chemical attacks^64,69^. The research presented in this manuscript began with the assessment of the aggressiveness of three selected factors: sulphuric acid, phenol, and urea. The analyses of aggressive solutions occurrence in the sewage infrastructure environment consider the concentrations that can exist in two different lines, i.e. for sewage and for sludge, and the fact that in the sewage line the treatment begins with raw sewage from sewage systems. The process of this treatment also includes three phases: mechanical purification, which is used to remove some of the organic substances that are deposited, a second phase, i.e. a biological process, which is carried out to remove organic substances, and a third phase, consisting of secondary sedimentation, which allows for a higher degree of purity. In each of these stages, the concentrations of chemical contaminants are different. In an anaerobic environment, bacteria present in waterways decompose fats, hydrocarbons and proteins because of chemical reactions, producing hydrogen sulfide, which then transforms into sulfuric acid. According to^71^, the presence of H_2_SO_4_ results from the activity of Thiobacillus bacteria in sewage water. As reported in^64,69^, bacterial metabolic reactions oxidize sulphides, elementary sulphur, and thiosulphates, producing sulphuric acid and lowering pH below 1. The sulphuric acid concentration can reach 5% or even 10%, with a pH of less than 1.

Phenol, mostly found in industrial or pharmaceutical wastewater, is less common in municipal sewage^72,73^. Due to its high aggressiveness toward various building materials, as described in the technical literature^72,73^, the effects of this substance on polyurea resin coatings were examined in the studies presented here. The concentration of phenol in wastewater treatment plants varies significantly depending on the type of wastewater (municipal or industrial) and the treatment stage. In Poland, average concentrations of phenols in wastewater flowing to municipal wastewater treatment plants usually range from 0.03 to over 100 µg/L, while in treated effluent wastewater they are much lower, ranging from 0.02 to about 10 µg/L. Phenol concentrations in industrial wastewater can even be much higher and depend on the industry.

The third compound selected for sewage system testing was urea, present in municipal wastewater treatment plants^74–76^. Past research indicates urea may also cause degradation of building materials^74–76^. Urea is an organic nitrogen compound that is hydrolyzed to ammonia (ammonium nitrogen) and carbon dioxide in municipal wastewater. For this reason, the concentration of total nitrogen or ammonium nitrogen is often reported in tests rather than urea alone. The concentration of nitrogen in the form of urea (converted to nitrogen) in wastewater directed to bioreactors in the tested treatment plants ranged from 9 to 14 µmol/L (micromoles of nitrogen per litre). In treated wastewater, this concentration is much lower and remains at a stable, low level, typically around 0.3 µmol/L (nitrogen micromoles per litre) or in the range of 0.7 to 2.4 µmol/L.

When deciding to test the products for their suitability in protecting concrete structures in the environment of municipal sewage treatment plants, we were guided by their chemical resistance, as indicated in the technical literature, a short application cycle on substrates, and rapid drying.

Research discussed in this study aimed to determine the following:

- whether polyurea resin coatings can effectively protect concrete from the harmful effects of selected groups of aggressive solutions found in domestic wastewater, together with the classification criteria for the resistance to the aggressive action of the above-mentioned groups of wastewaters,
- how the structure of the polyurea resin coatings alters after the referenced exposure,
- determine whether the potential damage to the polyurea resin coatings caused by service loads does not compromise their primary function, which is protecting concrete from the harmful effects of sulphuric acid, phenol, and urea solutions.

A novelty of the presented tests was the assessment of the durability of the mechanical properties of polyurea resin coatings operating in the sewage infrastructure environment to the action of selected aggressive environments (including sulphuric acid at 1% and 10%, phenol at concentrations of 0.1% and 1%, and urea at 3%), with particular emphasis on:

- An attempt at a mathematical classification of the degree of aggressiveness of the above interactions on polyurea resin coatings, considering the variability of susceptibility to degradation of the evaluated physico-mechanical properties within the population of polyurea resin coatings.
- The extension of the previously recommended assessment of resistance to chemical solutions, in relation to changes in external appearance and sometimes in adhesion, as required by EN 13,529^78^. Changes in other basic characteristics of mechanical properties, such as change in hardness, change in mechanical properties, or change in mass, were considered.
- Analyze the mechanism of mass change because of aggressive solutions, separating the problem of absorbability from the impact of actual chemical aggression.
- Different approach to assessing elongation. As a rule, elongation at break is typically evaluated, as seen in publication^77^. However, according to the authors of the manuscript, such results are irreversible and irreproducible because, after a decrease in the coating’s strength, they often transition into a state of flow. In connection with the above, the results discussed include the values of elongation at maximum tensile strength, which are considered repeatable and reproducible.

The article compares the mechanical properties of coatings with different thicknesses after exposure to aggressive solutions, whereas, as a rule, other publications present such properties for a single material. For example, in a very interesting publication^77^, the performance properties of the polyurea coating Master Seal M 686 were analyzed in detail, with a Shore A hardness of 92, tensile strength of 21 N/mm^2^, with elongation at break of 425%, but they were not compared with the properties represented by other materials.

A review of the literature on the subject suggests that no such attempts have been made so far. During the implementation of the discussed work, in addition to the already mentioned literature items, documents^64,69,71–80^ were used to determine the concentrations of chemical aggressive solutions used in the research, which resulted from long-term laboratory tests of chemical-resistant insulation made from resins. As can be seen from the above analysis, the values of aggressive solutions concentrations in the sewage infrastructure environment are variable and depend on several factors. They depend on both the location in the sewage infrastructure and the area from which the sewage is collected. Therefore, when assuming the concentration levels for the studies presented in the manuscript, the literature analyses presented above were guided by^64,69,71–80^ and our own previous research experience, including the assessment of the limit values declared by manufacturers of polyurea resin coatings for corrosion resistance under working conditions, as protection of concrete surfaces in environments exposed to chemical aggression^81^. Taking into account the above, the following concentrations were assumed in the studies for the three selected aggressive solutions: 1% and 10% for sulphuric acid, 0.1% and 1% for phenol, and 3% for urea. During the investigation, the so-called accelerated method was used. The principle of this method is that a higher concentration of aggressive solutions can be applied than naturally occurs in treated wastewater, allowing for a faster assessment of the effects of corrosion on polyurea resin coatings^2^.

## Materials

Based on the initial elimination tests conducted for various polyurea resin coatings, three commercially available coatings with different thicknesses starting from 1 mm to 3 mm and 5 mm and different initial mechanical properties typical of this group of materials, were selected for the ageing tests, i.e.:

- Product No. I − 3 mm thick grey coating, with an adhesion to concrete substrate of 2.3 MPa, Shore A hardness of 89, tensile strength of 20.5 MPa, and an elongation of 460%.
- Product No II – 1 mm blue coating with adhesion to concrete substrate of 3.3 MPa, Shore A hardness of 85, tensile strength of 13.6 MPa, and elongation of 120%.
- Product No III – 5 mm thick yellow coating, with adhesion to concrete substrate of 2.7 MPa, Shore A hardness 93, tensile strength of 28.2 MPa, at elongation of 367%.

Test samples were prepared by spraying and conditioned for 7 days under laboratory conditions, that is, at (21 ± 2)°C and relative humidity (60 ± 10)%, before conducting ageing tests. That’s possible because polyurea resin coating is a product of the reaction of two components, isocyanate and a resin mixture, and it exhibits an extremely short “tack-free” time^80^.

## Test methods

### Ageing exposure

Ageing tests aimed to identify the changes in polyurea resin coatings caused by exposure to the following chemically aggressive environments:

- A: 1% sulphuric acid (VI) solution.
- B: 10% sulphuric acid (VI) solution.
- C: Phenol solution with a concentration of 0.1%.
- D: Phenol solution with a concentration of 1.0%.
- E: Urea solution with a concentration of 3.0%.

**Fig. 1:**
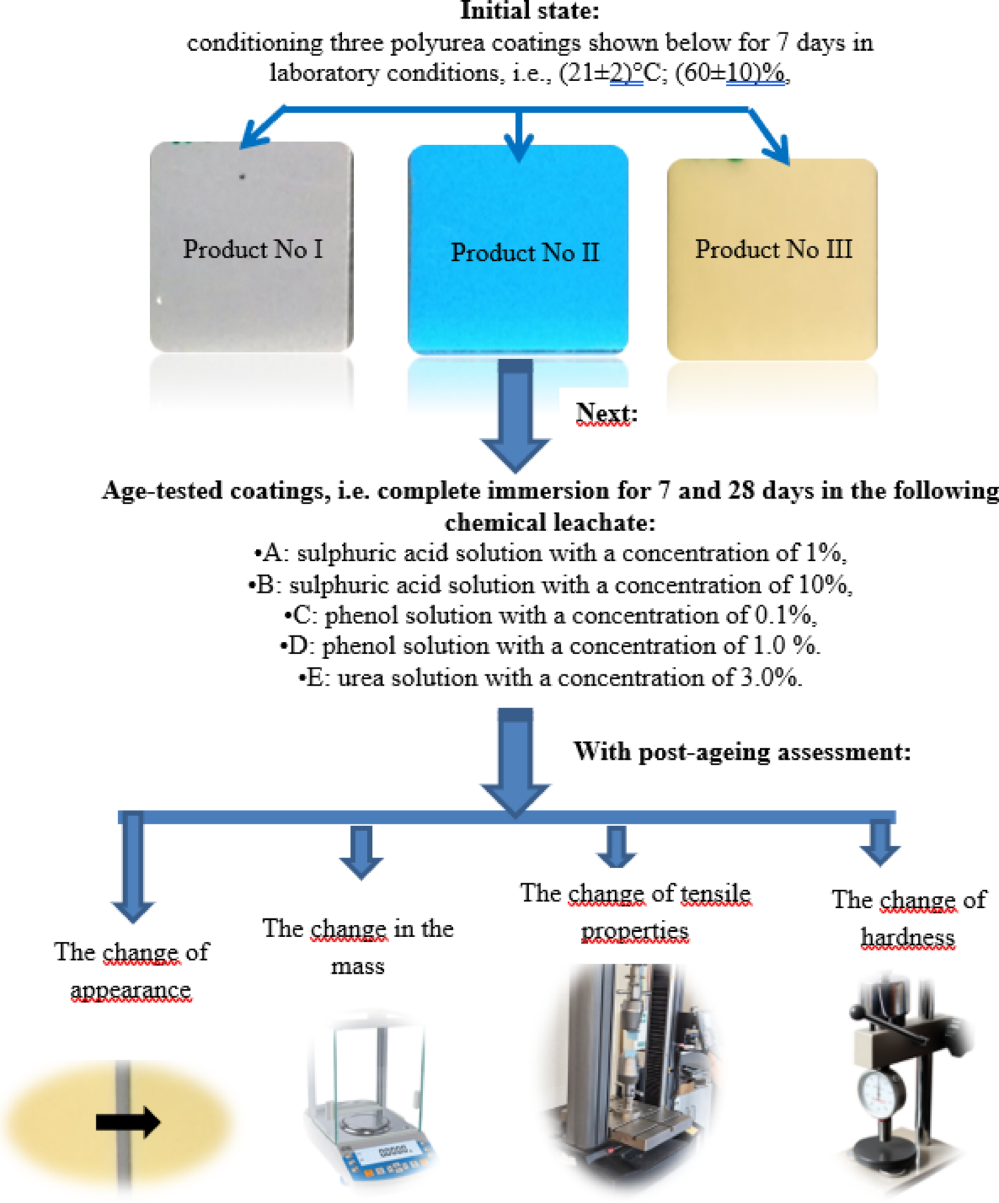
Experimental flowchart.

The concentration of solutions was intentionally set higher than what is usually found in nature. This was due to the artificial nature of the test, and to assess the effects of isolated solutions. The tests were conducted at an ambient temperature of (21 ± 2)°C. During testing, the samples were immersed in the solutions (keeping the solution level above and the samples about 10 mm high) without coming into contact with each other or the container walls. The polyurea coating was exposed to ageing solutions for 7 and 28 days, respectively. To avoid evaporation of liquids, the containers were tightly covered. After exposure, the samples were removed from the containers and subjected to further testing. The effects of ageing were measured by observing changes in appearance, mass, hardness, and tensile mechanical properties (Fig. 1).

An assessment of changes in external appearance after the action of chemical solutions was performed with the unaided eye, checking for possible discolouration, cracks, scratches, pitting, local cavities, and other mechanical damage visible on the surface of the coatings and on the edges of the sample.

### The change in the mass

The samples were placed in containers with covers holding 2.5 L of test solutions, allowing five 50 × 50 mm samples to be immersed for 7 and 28 days. Afterwards, the samples were removed from the solutions, dried on both sides with filter paper, and weighed within a maximum of 1 min with an accuracy of 0.001 g. Before exposing the samples to the ageing process, their mass was measured. For all tested samples, assessments were performed after exposure periods of 7 and 28 days, and after drying to a constant mass. Drying to solid mass was carried out at laboratory temperature, i.e. (21 ± 2)^o^C, weighing samples every 24 h. The weighing was completed when the next 3 weight measurements did not differ by more than 0.001 g. The mass loss (x) of the samples was calculated as a percentage using Eq. (1):

1 $$x=\left[ {\left( {{m_1}-{\text{ }}{m_0}} \right)/{m_0}} \right] \times 100\% ,$$

where:

-m_0_ is the constant mass and initial dry matter in grams,
-m_1_ is the sample weight after immersion or drying to a constant mass in grams.

The result was the arithmetic mean of the five tests.

### The change of hardness

The hardness test was performed using a Shore durometer–type A. It was conducted according to the test methodology specified in standard EN ISO 868:2003^82^ under standardised laboratory conditions on samples before ageing exposure and on test pieces after ageing exposure. The specimens were placed on a rigid horizontal surface, and the presser foot was positioned on the specimen’s surface. It was then pressed down to ensure firm contact without impact. A cone-shaped indenter made of hardened steel with a diameter of 1.25 mm ± 0.15 mm was then freely lowered onto the specimen, and after 15 s ± 1 s, the reading from the instrument was recorded to indicate the depth of penetration. Hardness is inversely related to the depth of penetration. The test result is the average of five measurements taken on each sample, with points at least 6 mm apart and at least 9 mm from the edge of the specimen.

### The change of tensile properties

The tensile properties of the coatings, both before and after the ageing process, were tested according to EN ISO 527-3:2018^83^ using a specimen type 5. Tests were performed on a computer-controlled Zwick strength testing machine with a 10 kN capacity head and an optical extensometer to measure elongation. The feed rate was set at 100 ± 10 mm/min. Tests took place under standardised laboratory conditions, specifically at (21 ± 2) °C and (60 ± 10) % relative humidity, on samples before ageing exposure and on test pieces after ageing exposure. Five samples were used for each series. Tensile strength, expressed in MPa, was calculated as the ratio of the maximum force to the sample’s cross-sectional area. Elongation was simultaneously monitored over a 25 ± 0.25 mm measurement section. The elongation value at maximum tensile strength was recorded as the initial value of the measurement section, and the relative elongation (in %) at maximum tensile strength was also determined.

### Mathematical assessment of coating degradation

The mathematical assessment of coating degradation due to environmental operation aimed to compare the effects of different aggressive solutions on coating properties. In the above assessment, two mathematical measures were used, i.e.:

- the Degradation Index (DI).
- the Integrated Resistance Index (IR ).

*The average DI – Degradation Index*, at which polyurea resin coatings degrade due to sulphuric acid, phenol, and urea is defined by Eq. (2).

2 $$\:\mathrm{A}\mathrm{v}\mathrm{e}\mathrm{r}\mathrm{a}\mathrm{g}\mathrm{e}\:\mathrm{d}\mathrm{e}\mathrm{g}\mathrm{r}\mathrm{a}\mathrm{d}\mathrm{a}\mathrm{t}\mathrm{i}\mathrm{o}\mathrm{n}\:\mathrm{r}\mathrm{a}\mathrm{t}\mathrm{e}\:\left(\mathrm{D}\mathrm{I}\right)=\frac{\varDelta\:m+\varDelta\:H+\varDelta\:w+\varDelta\:l\:}{4}$$

in which:

∆ m – change in mass, in %.

∆ H – change in hardness, in %.

∆ w – change in maximum tensile strength, in %.

∆ l – change in elongation at maximum tensile strength, in %.

The DI is the arithmetic mean of the percentage changes in the four key properties, measured after the same study period. When developing the DI indicator, it was considered that all four discussed changes in properties are significantly related to each other. The change in mass (understood as an increase in absorbency) significantly affects the change in the hardness of the tested products (the wet coating becomes softer). Upon further analysis, both above factors significantly affect the change in mechanical properties during tensile testing. Considering the above, all 4 properties were given equal importance in the development of the DI indicator. Although the individual properties have different physical units, the use of relative percentage changes allows them to be averaged without units, enabling the calculation of a comparable measure of the overall degradation of the material. The lower the DI value, the greater the total degradation impact of the environment.

The classification of the level of change in individual properties was adopted because of the percentage ranges observed in the studies, i.e.:

- change in the mass: low (< 5%), moderate (5–15%), high (15–25%), very high (> 25%),
- change of hardness: low (< 10%), moderate (10–15%), high (15–20%), very high (> 20%),
- change in maximum tensile strength: low (< 20%), moderate (20–40%), high (40–60%), very high (> 60%),
- change in elongation at maximum tensile strength: low (absolute change <|10%|), high (absolute change > |10%|).

The above classification levels were determined based on the requirements given in the technical literature for coatings intended for surface protection of concrete, respectively:

- In the case of mass change, the ranges referred to typical absorption levels of the aggressive medium, which in the literature are associated with swelling of coatings and secondary changes in mechanical properties, in particular hardness and elasticity^19,20,26^,
- The classification of hardness changes was based on criteria used in coating ageing studies, where decreases of 10–20% are the limit for maintaining mechanical resistance and resistance to operational damage^23,26^,
- The intervals of changes in tensile properties have been adopted in accordance with common practice in the analysis of the durability of polymeric materials and protective coatings, in which the reduction of mechanical properties by about 30–40% is treated as the limit of safe operation, while decreases exceeding 60% indicate advanced structural degradation of the material^19,20,65^.

*The Integrated Resistance Index (IR)* expressed on a scale of 0-100% was calculated as the arithmetic mean of the four sub-indicators, according to the Eq. (3):

3 $$RI=\frac{{{R_m}+{R_H}+{R_w}+{R_l}}}{4}\;\;[\% ]$$

in which:

*R*_*m*_- mass stability index: 100% - |∆*m*| (assuming that the change in mass is undesirable).

*R*_*H*_ - hardness change indicator: 100% + *∆ H* (assuming that *∆ H* is negative).

*R*_*w*_ - tensile strength index: 100% + *∆ w* (assuming that *∆ w* is negative).

*R*_*l*_ - elongation change rate at maximum tensile strength: 100% - *|∆ l* | (assuming that a large change is unfavorable).

The IR index takes values from 0% (total destruction) to 100% (no destruction).

*To validate the inference presented in the article*, an Analysis of Variance (ANOVA) was performed on the research results. This analysis aimed to determine whether there are statistically significant differences in the mean values and whether the variations in sample destruction caused by different factors (sulphuric acid, phenol, urea) are genuine and significant or merely due to random data scattering. The key value in this test is the p-value, which determines the probability of confirming or rejecting the correctness of the analysed test results; it is therefore the most important value in the calculation. When this value is less than 0.01%, it means that the presented results provide strong statistical evidence to reject the null hypothesis. In other words, we can say with almost 100% certainty that the type of chemical environment and exposure time are the factors that cause real and varied degradation effects in coatings.

## Results and discussion

### Physico-mechanical tests

**Fig. 2:**
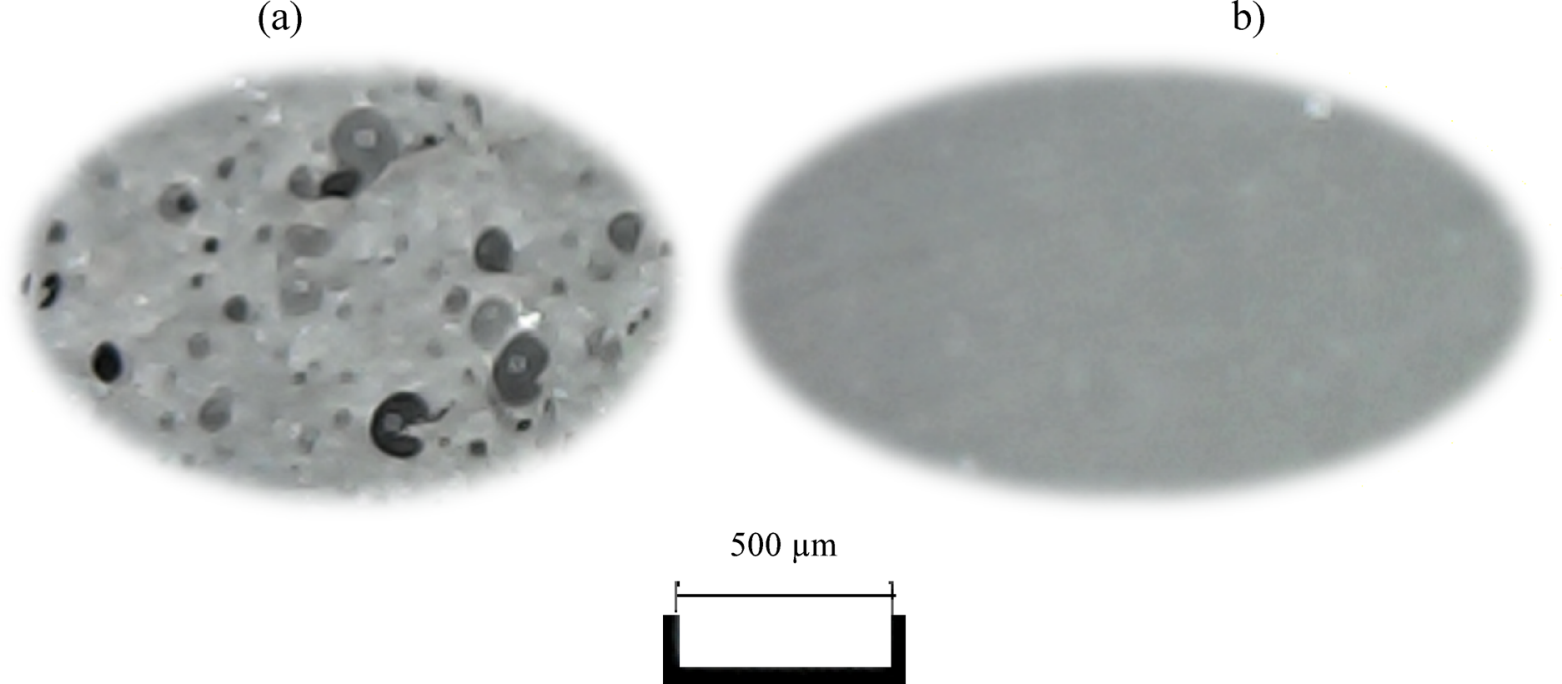
A 60-fold magnification of: (**a**) the cross-section, (**b**) the surface of the coating made of product no I before exposure to aggressive solutions (examination performed by Bresser USB Digital Microscope).

The evaluation of the resistance of polyurea resin coatings to selected environments found in municipal wastewater treatment plants was conducted using three randomly chosen products as examples. Figure 2 displays a 60-fold magnification of the cross-section and surface of the coating made of one of them, i.e. from product No. I, before exposure to aggressive solutions. The coating is continuous, and the material’s structure is compact. The nanostructure of the coating contains microscopic pores that differ in size and distribution. Both on the surface and in the cross-section, the fillers are visible, and their distribution is uniform.

Given the significance of sulphuric acid, phenol, and urea, the coatings were exposed to solutions with the following concentrations: sulphuric acid at 1.0% and 10.0%, phenol at 0.1% and 1.0%, and urea at 3%.

**Fig. 3:**
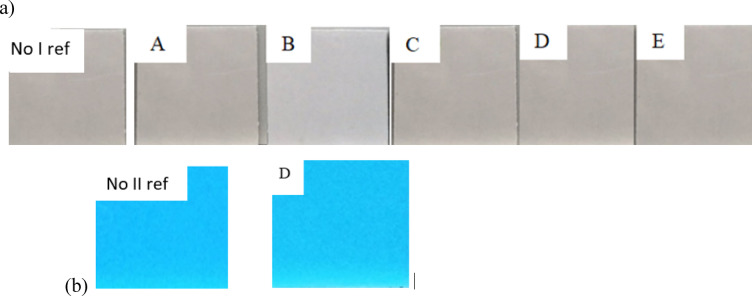
Examples of the appearance of coatings made from products: I (point a) and II (point b). The symbols: No I ref and No II ref are used to designate reference samples (i.e. before the ageing factors) and the symbols A, B, C, D i E after interaction in selected environments respectively: A − 1% sulphuric acid, B − 10% sulphuric acid, C − 0.1% phenol solution, D − 1% phenol solution, E- 3% urea solution.

**Fig. 4:**
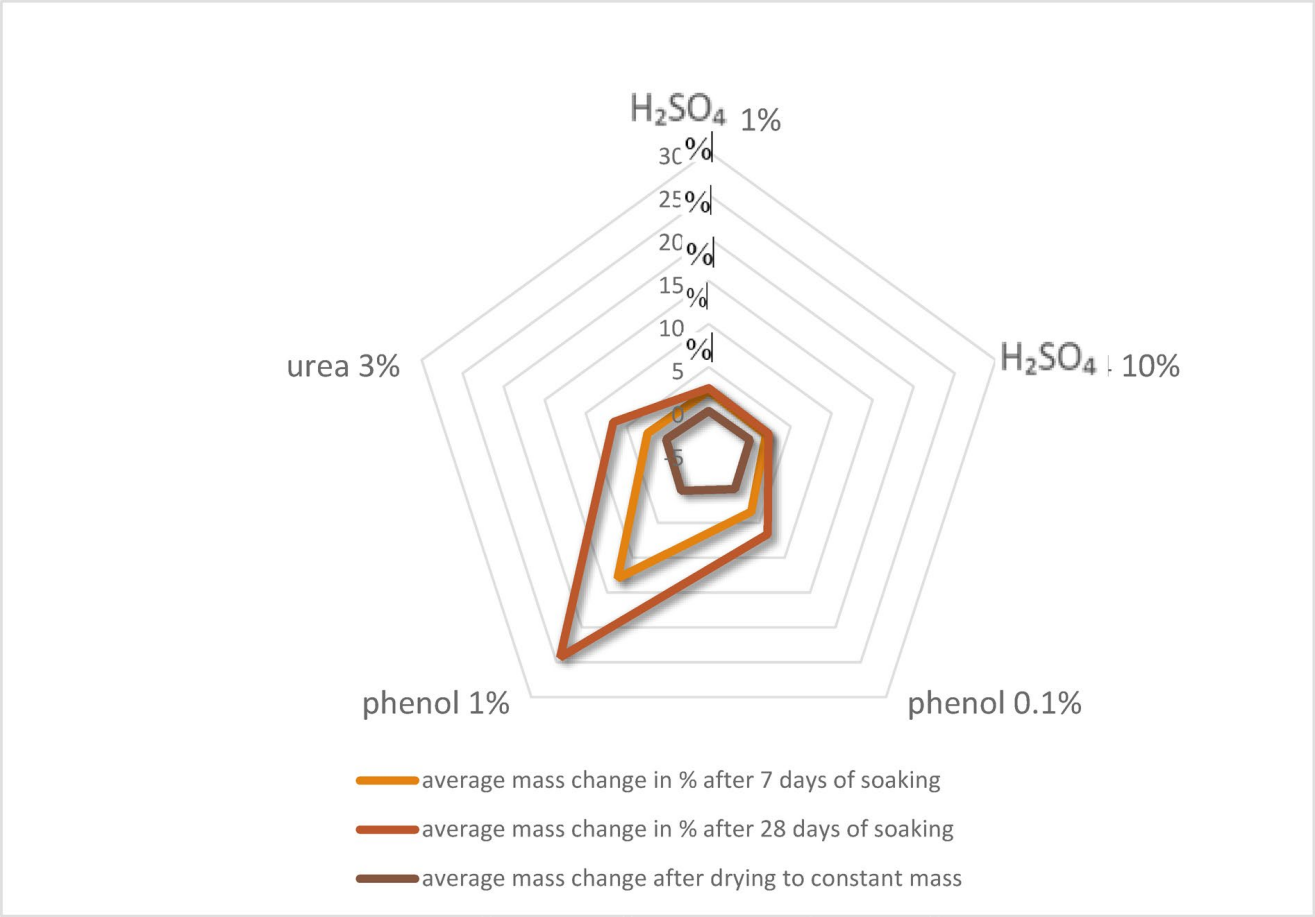
Average mass change of polyurea resin coatings I, II, and III in %, after 7 days, 28 days of immersion, and after subsequent drying.

**Fig. 5:**
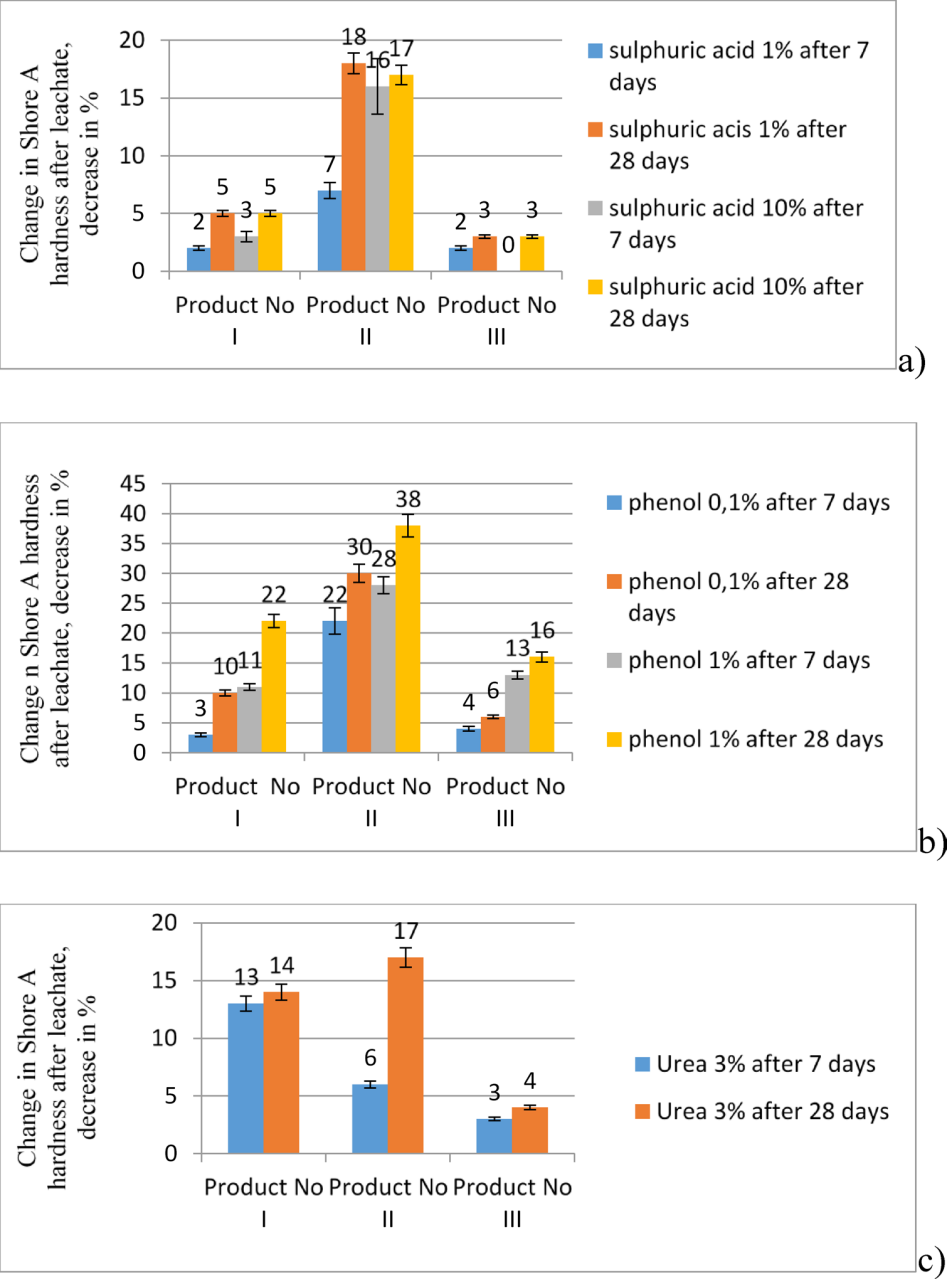
Changes in % of Shore A hardness values due to aggressive solutions after 7 and 28 days of treating polyurea resin coatings I, II, and III in: (a) sulphuric acid solutions, (b) phenol solutions, (c) urea solutions; Error bars are additionally marked in the drawings.

**Fig. 6:**
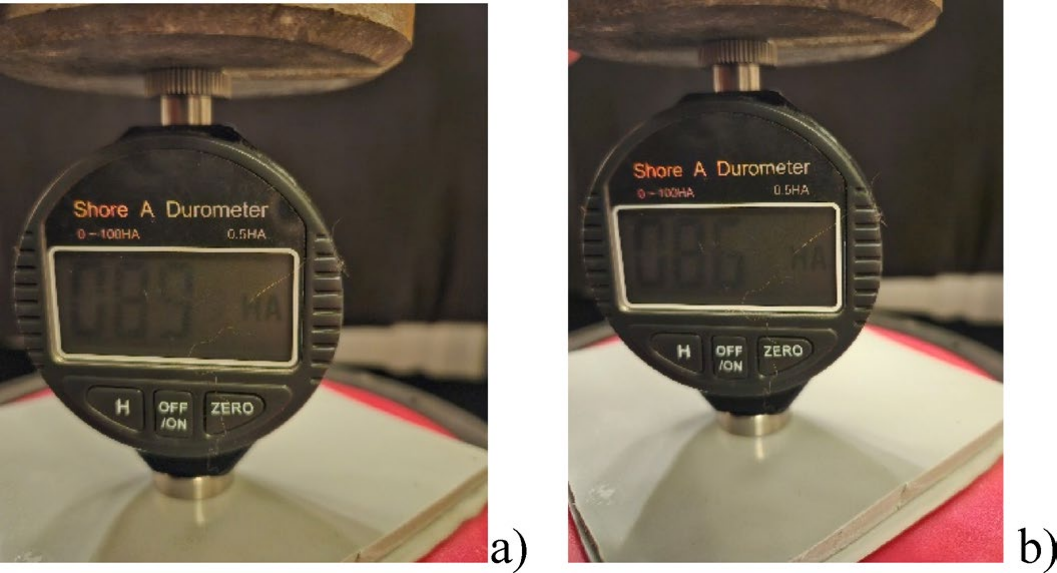
Sample of measurement of the Shore A hardness of product no I: (a) before aging, (b) after 28 days of exposure to 10% sulphuric acid.

**Fig. 7:**
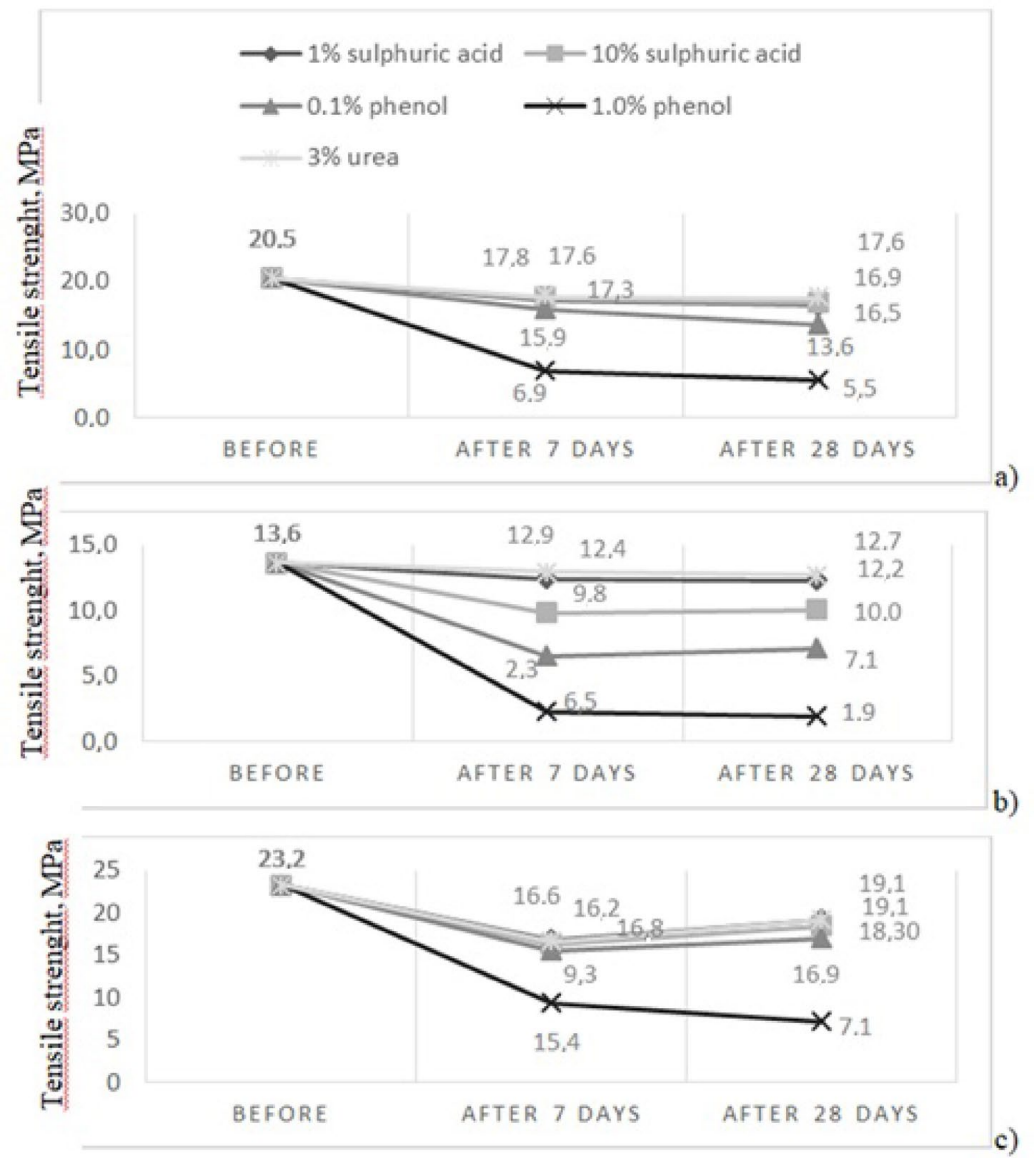
Tensile strength at break during testing (average values): (a) product No I, (b) product No II, (c) product No III.

The results of the tests assessing the impact of these factors on the properties of the tested products are presented in the following tables and figures:

i. Table 1 summarises the results of changes in the external appearance of the tested coatings and additionally shows them in Fig. 3.
ii. Table 2 shows the results of mass changes, also displayed in Fig. 4.
iii. Figure 5 show Shore A hardness results after exposure, Fig. 6 shows the measurement of the hardness of product no I: sample before aging, after 28 days of exposure to 10% sulphuric acid and after drying to solid mass.
iv. Table 4 shows changes in tensile strength after exposure. Figure 7 graphically illustrates the changes in the mechanical properties of the three tested products after applying the aforementioned factors during tensile testing.

**Table 1:** The changes in appearance after environmental influence.

Product *n*^o^	Sulphuric acid	Sulphuric acid		0.1% phenol	1.0% phenol	3.0% urea
	1.0%	10.0%				
I	No change		Noticeable yellowish discoloration	No change	No change	No change
II	No change		No change	No change	Noticeable loss of gloss	No change
III	No change		No change	No change	No change	No change

Figure 3 shows:

- the appearance of an example of one coating (product No I) by comparing the changes in appearance after 28 days of its immersion in solutions: sulphuric acid with concentrations of 1.0% and 10%, phenol with concentrations: 0.1% and 1% and urea with a concentration of 3% in relation to the sample before the interactions, and.
- coatings from product no. II – after the action of a phenol solution with a concentration of 1.0% in relation to the sample before the interactions.

The above examples are limited to the specific cases mentioned. In the absence of clear damage to the coatings, the sample photos can only be treated as illustrative, as they do not significantly impact the discussion of the results.

Tables 2, 3 and 4 show the average changes in performance obtained for each of the 3 tested products. The results in the last two columns of each table were supplemented with the average value calculated based on data for three different coatings, along with an additional assessment of this mean using the coefficient of variation. This value is treated as the achievable average change for the population of randomly selected polyurea resin coatings exposed to the investigated aggressive solutions and, together with the coefficient of variation characterizing it, provides information on the possible, expected limits of changes for the population of the above-mentioned products. Unfortunately, for a population consisting of 3 representatives, the coefficients of variation signal significant possible dispersions of the discussed values of changes in a few cases but nevertheless allow for obtaining an exemplary picture of the variability resulting from the studied interactions.

**Table 2:** Percent change in mass of polyurea resin coatings after exposure to aggressive agents.

Environment	Stage of study, after	Average value of change in mass for each coating, accordingly for samples: % m/m / standard deviation			Average value of change for three coatings	
		I	II	III	Population average, % m/m	Coefficient of variation,%
1% H₂SO₄	7 days	2.0 / 0.2	2.8 / 0.1	1.9 / 0.0	2.23	20.2
	28 days	2.4 / 0.1	3.0 /0.1	2.2 / 0.1	2.53	15.8
	drying	-0.1 / 0.0	0.1 / 0.0	-0.1 /0.0	-0.03	0.8
10% H₂SO₄	7 days	1.8 / 0.1	2.5 / 0.1	1.7 / 0.0	2.00	20.0
	28 days	2.1 / 0.3	2.8 / 0.3	2.0 / 0.1	2.30	19.1
	drying	-0.1 / 0.0	0.2 /0.0	0.0 / 0.0	0.03	1.5
0.1%Phenol	7 days	2.9 / 0.3	4.7 / 0.4	2.7 / 0.2	3.43	30.3
	28 days	5.9 / 0.4	9.2 / 0.6	4.8 /0.5	6.63	33.5
	drying	0.0 /0.0	0.4 /0.0	0.1 /0.0	0.17	2.9
1% Phenol	7 days	12.1 /0.5	14.9 / 1,0	11.9 / 0.8	12.97	12.1
	28 days	18.5 / 0.5	29.1 /2.1	25.3 / 3.1	24.30	22.2
	drying	0.0 / 0.0	1.4 / 0.0	0.1 / 0.0	0.5	4.1
3% Urea	7 days	2.0 / 0.1	3.1 / 0.1	2.3 / 0.1	2.47	22.3
	28 days	7.5 / 0.6	6.9/ 0.1	5,5 / 0,5	6.63	15.7
	drying	0.2 /0.0	0.0 /0.0	0,4 / 0,1	0.20	2.7

**Table 3:** Changes in tensile strength of polyurea resin coatings after exposure to aggressive agents.

Environment	Stage of study, after days	Average value of change in tensile strength for each coating, accordingly for samples, %/ / standard deviation			Average value of change for three coatings	
		I	II	III	Population average, %	Coefficient of variation,%
1% H₂SO₄	7	-15 / 2	-18/ 2	-10 / 1	-14.33	28.2
	28	-20 / 2	-25/ 3	-15 / 3	-20.00	25.0
10% H₂SO₄	7	-15/ 1	-20 / 2	-12 / 2	-15.67	25.8
	28	-25 / 3	-28/ 3	-20 / 2	-24.33	16.6
0.1%Phenol	7	-40 / 3	-45 / 5	-35 / 5	-40.00	12.5
	28	-65 / 5	-70 / 8	-60 / 4	-65.00	7.7
1% Phenol	7	-70 / 8	-75 / 6	-65 / 5	-70.00	7.1
	28	-80 / 7	-85 / 9	-78 / 2	-81.00	4.3
3% Urea	7	-12 / 1	-15 /3	-10 /1	-12.33	20.4
	28	-25 / 3	-28 / 4	-28 / 4	-25.00	12.0

**Table 4:** Changes in elongation at maximum tensile strength of polyurea resin coatings after exposure to aggressive agents.

Environment	Stage of study, after days	Average value of change in elongation at maximum tensile strength for each coating, accordingly for samples, %			Average value for three coatings	
		I	II	III	Population average, %	Coefficient of variation ,%
1% H₂SO₄	7	0.4/ 0.0	-1.7 / 0.2	3.3/ 0.4	0.7	6.3
	28	-6.9/ 0.1	10.6 / 2.4	-0.3/ 0.0	1.1	78.1
10% H₂SO₄	7	-0.7 /0.2	5.8 /0.5	-3.5 /0.4	0.5	22.8
	28	− 7.8 / 0.5	6.2 / 0.8	-5.2/ 0.6	-2.3	55.5
0.1%Phenol	7	-0.7 /0.0	10.2 / 1.5	-0.3/ 0.0	3.3	35.9
	28	-0.9/ 0.1	10.4 / 1.1	4.9 / 0.3	4.8	31.9
1% Phenol	7	-28.5 / 2.1	1.7 / 0.1	-7.1 /1.1	-11.3	241.2
	28	-34.5 / 3.3	-10.8 / 0.3	-24.3 / 3.1	-23.2	141.3
3% Urea	7	-0.5/ 0.0	5.0 / 0.6	-4.0 / 0.4	0.2	20.6
	28	-8.0 / 1.1	4.0/ 0.2	-6.0 / 0.6	-3.3	41.3

After 28 days of soaking the samples in sulphuric acid (1% and 10%), phenol (0.1% and 1.0%), and 3% urea, no noticeable changes were observed in their appearance. No surface damage, such as cracks, blisters, or spalling, was observed. The coatings’ surfaces remained smooth. The only change noted, relevant for utility, is a local yellowish discolouration for product No. I, after exposure to a 10% sulphuric acid solution, and loss of gloss on the surface of product No. II after exposure to a 1.0% phenol solution. This may indicate that aggressive environments have a greater impact on the ageing processes within the polyurea resin coatings. However, the results for product No. III challenge this idea, as no appearance changes were observed after exposure to aggressive solutions. Summing up in all the cases studied, after removing the samples from the test solutions, no mechanical damage was found during the evaluation of their external appearance, and only in two cases a slight loss of gloss or slight discoloration. Thus, the absence or appearance of slight changes in colour or loss of gloss, if they are not accompanied by visible mechanical damage, does not significantly affect the changes in the mechanical properties of the coatings. This is confirmed by observations not only of product III, but also of products I and II in general, in which, after the action of most of the tested aggressive solutions, only two variants were found to lose gloss and a slight change in colour. At the current stage of the study, it is challenging to determine whether major changes in external appearance, such as those caused by UV radiation, will impact their mechanical properties, as the studies presented in this manuscript did not account for such exposures. According to literature reports, polyurea resin coatings^65–68,77^ are not resistant to UV radiation, which can cause significant discolouration/loss of colour.

After drying the coatings to a constant mass, no surface damage was observed in any of the investigated cases, including cracks, blisters, or spalling, with no colour change.

The external appearance assessment of the coatings after exposure to these aggressive solutions was supplemented with weight change measurements. An apparent increase in weight was observed during the first 7 days of soaking in both 1% and 10% sulphuric acid solutions, with smaller increases after an additional 21 days. The mass increase immediately after removal from the liquid was approximately 2–3%, likely due to saturation, as the weight change after drying fell within the measurement error. In the case of phenol and urea solutions, mass changes are significant after removal from the liquid but return to initial values after drying. Phenol 0.1%, after 7 days of soaking, causes an increase in moisture content of 3 to 5%, and rises from 5% up to 9% after 28 days. Even greater weight gains are observed with 1% phenol solution, from 12 to 15% after 7 days, and from 19 to 29% after 28 days. After 7 days of soaking in 3% urea solution, weight gains range from 2 to 3%, increasing from 5% to 7% after 28 days. In all tested cases, once dried to a constant weight, the samples returned to their original values; thus, no leaching was observed due to the test solutions. However, it should be noted that under operating conditions at wastewater treatment plants, these coatings can be continuously exposed to aggressive solutions without intermediate drying. Therefore, it is essential to evaluate whether the coatings’ vulnerability to mechanical damage increases when wet, for example, due to a possible loss of hardness and increased susceptibility to impact from hard impurities in the wastewater.

The results of the Shore A hardness test also show that phenol solutions have the most significant effect on all polyurea resin coatings, especially at a concentration of 1%. The greatest reduction in hardness after phenol treatment was seen in the thinnest layer, which also had the highest absorption values and the largest decreases in strength, despite initially having the lowest of these parameters. This could threaten the coating’s integrity under service load conditions. Depending on the product, polyurea resin coatings with compressive strengths below 20 MPa and elongations of not more than 300% may not be suitable for use in environments containing phenol, sulphuric acid, and urea, even if they adhere well to the substrate. There is a clear tendency to keep slight changes in hardness after treatment with sulphuric acid, regardless of whether the concentration is 1% or 10%. On the other hand, urea causes unclear changes in both hardness and weight. No definitive pattern was observed regarding its negative effects on coatings, their thickness, and tensile mechanical properties. Clearly, increasing the saturation of coatings in a 3% urea environment can result in a significant loss of hardness, even in coatings with substantial thickness.

There were decreases in the tensile strength of the tested coatings across all environments tested, but the highest values were observed with 1% phenol exposure, reaching up to 80% of the original values. It can be assumed that, in cases like these, the length of the soft segment of polyurea responsible for its properties under dynamic conditions decreases, as reported in the literature^84^. The relative elongation was found to increase proportionally with longer soft segments, along with a corresponding decrease in tensile strength. A high coefficient of variation in the elongation at break results indicates low repeatability within this study. After exposure to sulphuric acid and urea, the reductions in tensile strength are notably lower, ranging from 10% to 30%, regardless of the agent’s concentration, while the elongation of the tested coatings remains unchanged. A decrease in tensile strength and elongation at the maximum strength of the tested coatings is associated with a gradual decrease in their elasticity, which, in consequence, may contribute to the formation of microcracks and other surface damage under operating conditions. According to the authors of the manuscript, the permissible limits for these functional properties have not yet been determined. However, when comparing the above-mentioned products with other materials used to protect concrete surfaces, including waterproofing materials, values at 40% of the initial values are assumed to be safe limits, especially since the breaking of the reference strips of polyurea resin coatings in the tensile process is typically fragile.

In summary, the research findings suggest that the analyzed chemical environments could cause the following changes in the properties of polyurea resin coatings:

- The sulphuric acid solutions at 1% and 10% concentrations are the least aggressive among the three tested solutions. Increasing the concentration from 1% to 10% does not lead to a significant increase in damage and results in:

- A slight increase in coating weight due to moisture (ranging from 2% to 3%), along with a simultaneous decrease in hardness, reaching up to 10%.
- There is a slight decrease in tensile strength, ranging from 10% to 30%, along with a small change in elongation at maximum tensile strength, with maximum drops of up to 2.5% and increases of up to 0.5%.

(b) A 3% urea solution causes more notable changes in coating properties than sulphuric acid solutions, but these changes should not result in significant damage. The main factor is a reduction in hardness by up to 13%, making the coating softer and more susceptible to potential mechanical damage from hard elements in the wastewater. Naturally, the decrease in hardness correlates with an increase in coating weight of about 5 to 7%. The impact on tensile strength and elongation at maximum force from this solution is similar to that caused by sulfuric acid solutions.
(c) Polyurea resin coatings have the lowest resistance to phenol. Even a slight increase in the concentration of this agent from 0.1% to 1% results in significant changes in coating resistance, turning phenol from a moderately harmful substance into a destructive one, and causing, respectively:

- increase in weight from 9% to 30%, while the average decrease in hardness occurs from 17% to 27%.
- A significant decrease in tensile strength occurs from 60% to 80%, with little or only a slight increase in elongation at maximum tensile strength after exposure to a 0.1% solution (up to about 5%). However, exposure to a 1% solution causes a drastic decline in this parameter, reaching as high as 23%.

### Mathematical assessment of the ageing rate of polyurea resin coatings as a result of aggressive solutions action

**Fig. 8:**
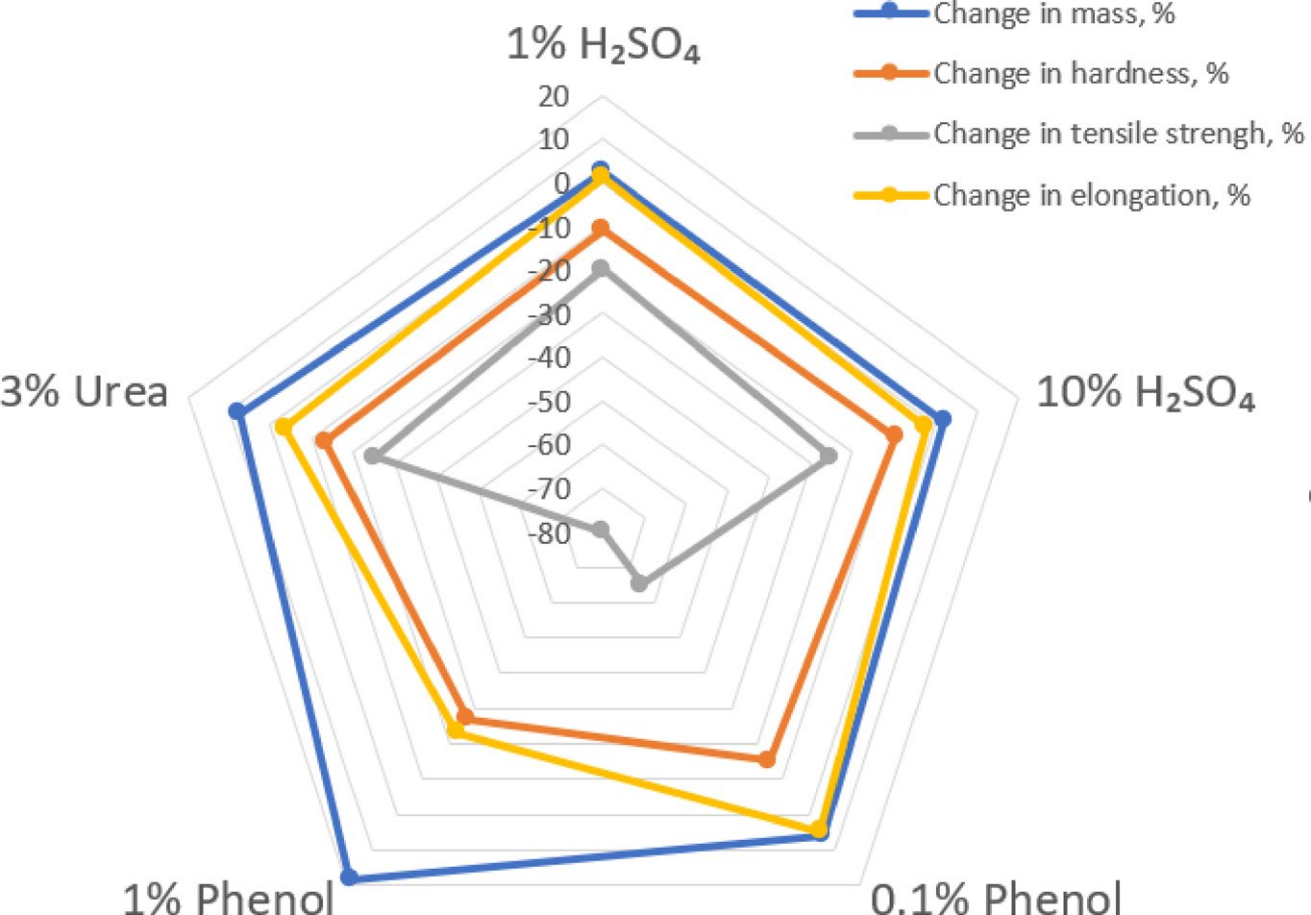
Changes in the investigated properties of polyurea’s coatings resulting from treatment with sulphuric acid (1% and 10%), phenol (0.1% and 1%), and 3% potency solutions.

The ranking of polyurea resin coatings’ resistance, based on the calculated degradation index in selected aggressive environments, is shown in Table 5; Fig. 8. The lower the DI value, the greater the total degradation impact of the environment.

However, it should be emphasized that equal weights of the DI indicator represent a certain simplification of the method. Under real-world operating conditions, the meaning of each property may vary depending on the design function of the coating and the nature of the chemical environment. In future studies, we will consider differentiated weights, e.g. based on reliability analysis, operational damage analysis or multi-criteria methods (MCDA), which would allow for further improvement of the proposed DI.

**Table 5:** Ranking of polyurea resin coatings’ resistance to specific aggressive environments.

Aggressive agent	Mass change (↑)	Hardness loss (↓)	Tensile strength loss (↓)	Elongation, change	Overall degradation (DI)	Resistance rank
H₂SO₄ (1–10%)	Low (2 ÷ 3) %	Low (~ 10%)	Moderate (10 ÷ 30) %	Low (-2.5 ÷ 0.5) %	**-8.1/ Low**	The Best
Phenol 0.1%	Moderate (5 ÷ 9) %	High~ 16.8%	High (40 ÷ 60) %	Low (3.3 ÷ 4.8) %	**-12.4/High**	
Phenol 1%	Very high (20 ÷ 30) %	Very high (~ 27.1%)	Very high (up to 80%)	High (-11.3÷-23.2) %	**-31.9/Very high**	The worst
Urea 3%	Moderate (5 ÷ 7) %	High (~ 13.1%)	Moderate (10 ÷ 30) %	Low (-3.3 ÷ 0.2) %	**-9.7/ Medium**	

The average changes in the properties of the studied polyurea’s coatings caused by exposure to solutions of sulphuric acid with concentrations from 1% to 10%, phenol at 0.1% and 1%, and urea at 3% are shown graphically in Fig. 8. The radar graph (Fig. 8) clearly shows that polyurea resin coatings have strong resistance to acidic environments, while phenol causes significant decreases in all tested properties. In the case of urea, the damage is slightly more severe than that from sulphuric acid but significantly less than from phenol solutions.

**Fig. 9:**
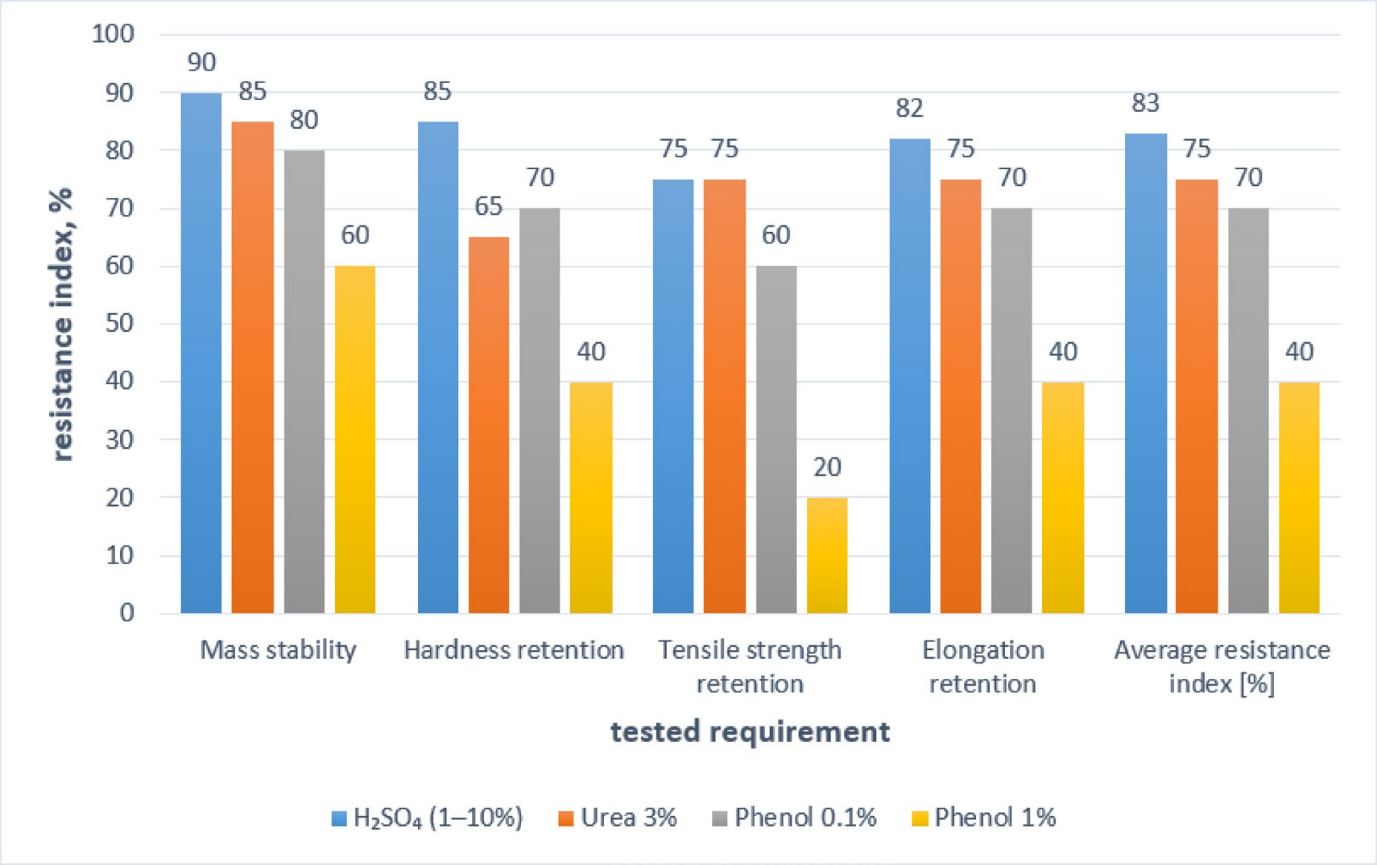
Percentage of tested chemical solutions on selected properties of polyurea resin coatings.

The average resistance index of polyurea’s coatings to selected environments, based on four key properties of the tested products, is shown in Table 6 and illustrated in Fig. 9. The ranking system uses a scale of 0 to 100 points, with 100 indicating the highest resistance and 0 the lowest.

**Table 6:** Classification of polyurea resin coatings resistance to specific chemical environments, based on four key properties.

Aggressive agent	Mass stability	Hardness retention	Tensile strength retention	Elongation retention	Average resistance index [%]
H₂SO₄ (1–10%)	90	85	75	82	**83**
Urea 3%	85	65	75	75	**75**
Phenol 0.1%	80	70	60	70	**70**
Phenol 1%	60	40	20	40	**40**

In summary, the above research findings suggest the following statements regarding the suitability of polyurea resin coatings for sewage infrastructure facilities:

- The coatings are highly resistant to sulphuric acid solutions with concentrations ranging from 1% to 10%, making them effective in protecting concrete under such conditions.
- Monitoring the environment of a 3% urea solution or a 0.1% phenol solution involves inspecting the coating condition, mainly for loss of hardness.
- In an environment with a 1% phenol solution, polyurethane coatings will degrade rapidly and are therefore not recommended for protecting concrete in such conditions. The authors suggest exploring other specialized solutions for these applications.

The results of an Analysis of Variance (ANOVA) are shown in Table 7.

**Table 7:** Variance analysis of the research results presented in the article.

Sources of volatility	Sum of squares (SS)	Degrees of freedom (df)	Medium Square (MS)	F Stat (F)	*P*-value
Between groups	2450.82	14	175.06	42.15	< 0.0001
Inside groups	124.58	30	4.15	-	-
Sum	2575.40	44	-	-	-

Between groups – variability caused by differences among factors (acid vs. phenol vs. urea).

Within groups – variability caused by, for example, measurement error, differences between samples, and other errors (“random noise”).

Sum – the total amount of volatility observed in all data.

Sum of squares – a measure of total volatility, 2450.82 > 124.58, or various factors. (acid vs. phenol vs. urea) have a greater impact than “noise”.

Degrees of freedom – the number of independent data points used to calculate the estimators (5 factors x 3 measurement times).

Mean square – variance (SS : df).

The obtained F-statistic value is 42.15, which is very high. This indicates that the differences among the interactions with acid, phenol, and urea are significantly greater than what could be attributed to “random noise,” i.e., variability resulting from measurement error, differences between samples, and random error. The p-value, the most critical measure in the calculation, indicates the probability of the test result and is less than 0.0001. This means the result is statistically significant, allowing us to reject the null hypothesis (i.e., no effect). A p-value below 0.0001, well below the conventional significance levels (α = 0.05 or α = 0.01), suggests that the probability the observed dramatic differences between groups (environments) are due to chance is less than 0.01%. Therefore, we can confidently state that the type of chemical environment (acid, phenol, urea) and exposure time are the real factors causing varying degradation effects in coatings. This statistical result confirms all observations made from the research. Differences that appeared obvious, such as the significant effect of a 1% phenol solution versus the minimal effect of sulphuric acid, are validated by this rigorous statistical test. The differences in degradation levels caused by acid, phenol, and urea are statistically significant and genuine.

## Conclusions

The article reports the results of a study on how specific chemical solutions used in conjunction with concrete structures in municipal wastewater treatment plants affect the durability of certain properties of polyurea resin coatings used for surface protection on these substrates. The study tested solutions including sulphuric acid at concentrations of 1% and 10%, phenol at concentrations of 0.1% and 1%, and urea at a concentration of 3%. Based on the findings, the following conclusions can be drawn:

- The average values clearly confirm the established order of aggressiveness among the tested environments, ranking them from the most to the least aggressive: 1% Phenol > 0.1% Phenol > 3% Urea > 10% H₂SO₄ > 1% H₂SO₄. Phenol 0.1% contributes to greater changes in properties, such as weight, hardness, tensile strength, and elongation, compared to urea 3%. The aggressiveness of phenol increases significantly with its concentration, resulting in drastic changes in the above properties at a 1% concentration of the solution.
- A clear, progressive decline in all properties is seen as exposure time increases from 7 to 28 days across all environments. The degradation process does not stabilize within the initial period of environmental impacts, that is, after 7 days.
- The research suggests that polyurea resin coatings are likely suitable for environments containing sulphuric acid. Changes in weight, hardness, and tensile properties are minimal across solutions with concentrations from 1% to 10%, with tensile strength dropping by only 10–30% and hardness decreasing by up to 10%.
- In large-scale sewage environments, such as those involving urea solution (3%), a hardness loss of up to 13% should be considered. This can contribute to damage from abrasion or impacts by hard particles in wastewater. The environment described above does not cause a significant reduction in the tensile strength of the tested coatings, which remains within the range of 10–30%, like the effect of a sulphuric acid solution.
- In a phenol environment, it is highly recommended to exercise extreme caution when considering the use of polyurea resin coatings for protecting structures. This environment poses the greatest risk to the durability of these products, especially at a 1% concentration, which significantly increases the coatings’ absorbency (by up to 30%), reduces their tensile strength (by up to 80%), and lowers their hardness (by up to 27%). Therefore, choosing the highest quality products (such as product No. III) and performing regular inspections of the facilities’ technical condition are essential. Under these conditions, such coatings are not advised.
- The increase in the coating’s weight immediately after removal from the tested liquids is due to moisture content, as the weight returns to its original level after drying. However, it is important to remember that in sewage treatment plant conditions, these coatings may be constantly exposed to aggressive solutions without any drying breaks.
- Polyurea resin coatings with tensile strengths below 20 MPa and elongations below 300% may not be suitable for use in environments containing phenol, sulphuric acid, and urea, even if they exhibit high adhesion to the substrate.
- Differences in the degradation levels of polyurea resin coatings across various chemical environments were confirmed through analysis of variance. A p-value of less than 0.0001 (p-value), which is well below standard significance levels, indicates that the result is statistically significant and allows rejection of the null hypothesis (e.g., no effect), suggesting that the observed phenomenon is unlikely due to chance. The probability that the significant differences observed between groups (environments) are due to chance is less than 0.01%.

Summarising the results of the research presented in this manuscript, it can be concluded that when designing chemically resistant protection of concrete structures operating in the environment of sewage infrastructure, the choice of polyurea resin coatings seems to be a solution:

- safe in the case where sulphuric acid with a concentration of up to 10% is expected in aggressive solutions,
- not recommended in the phenol environment, especially at higher concentrations of 1%. The use of such coatings in this case is associated with a very high risk of complete loss of protective properties in a relatively short time,
- requiring additional analysis of the structure of the wastewater containing urea, with particular emphasis on the sludge line. A significant loss of hardness in the coatings under the influence of such an environment may result in mechanical damage due to accelerated abrasion.

As part of the research described in this manuscript, important classification criteria for the resistance of polyurea resin coatings to the aggressive action of selected wastewater groups have been established, representing a significant technical innovation in this field of knowledge. This enabled us to identify directions for further investigation of the described problem. In the next phase, it is planned to determine the causes of the observed phenomena and the effects of their action, with particular focus on the microstructure of the coating and the substrate beneath it, including the adhesion zone and the physicochemical transformations observed during exposure. The planned research also considered it useful to determine whether the damage process described in this manuscript stabilizes over a longer period in aggressive environments.

Due to the variety of interactions in the sewage infrastructure environment, depending on the type of conditions received, and the short exposure time of the samples, the technical data presented in the manuscript can be considered significant trends indicating possible threats to the durability of polyurea resin coatings as a protection for concrete surfaces.

## Data Availability

All data generated or analysed during this study are included in this published article.
